# Protecting strength and integrity in virology

**DOI:** 10.1128/jvi.00890-26

**Published:** 2026-08-26

**Authors:** Paul D. Bieniasz, Charles M. Rice, Terence S. Dermody, Lynn W. Enquist, Felicia Goodrum, David B. Kushner, Helen M. Lazear, Stanley M. Lemon, Andy Pekosz, J. Purdy, Vincent R. Racaniello, Linda J. Saif, Tony Schountz, Susan R. Weiss, Christiane E. Wobus, James Alwine, Stefano Bertuzzi, Jeanne Marrazzo, Shan-Lu Liu

**Affiliations:** St Jude Children's Research Hospital, Memphis, Tennessee, USA

**Keywords:** ASV, ASM, IDSA, debarment, Ralph Baric

## Abstract

The American Society for Virology (ASV), American Society for Microbiology (ASM), and the Infectious Diseases Society of America (IDSA) express concern that the proposed suspension and debarment of Dr. Ralph Baric, in the absence of publicly presented evidence of research misconduct or noncompliance with applicable regulations, could undermine scientific integrity, virology research, and U.S. preparedness for future infectious disease threats.

## EDITORIAL

The American Society for Virology (ASV), American Society for Microbiology (ASM), and the Infectious Diseases Society of America (IDSA) are alarmed by the initiative of the U.S. Department of Health and Human Services (HHS) to suspend and debar from federal funding Dr. Ralph Baric (1). Attacks on virology and virologists undermine pandemic preparedness and weaken our ability to respond to emerging infectious diseases.

Our organizations support national and international cooperation to ensure that virology research and public health measures are conducted with appropriate oversight to maintain safety and public trust. We are unaware of any evidence that Dr. Baric’s studies were conducted outside the research guidelines and oversight frameworks in place at the time, and his work was approved by the National Institutes of Health and the relevant institutional biosafety committees.

We believe that Dr. Baric’s scientific record is clear. He is an internationally respected virologist whose work has focused on understanding virus replication and pathogenesis, as well as preventing or mitigating viral outbreaks. His contributions have been recognized by election to the National Academy of Sciences and the American Academy of Arts and Sciences. In addition, Dr. Baric is a highly regarded mentor who has trained generations of scientists committed to rigorous experimentation conducted under appropriate biosafety conditions.

ASV, ASM, and IDSA recognize and value Dr. Baric’s scientific contributions and believe that actions of this magnitude must be evidence-based and conducted with integrity and transparency. Efforts lacking transparency and clear evidence risk harming individual scientists as well as the broader U.S. scientific enterprise.

## EDITOR'S NOTE

**Ed. Note**: This editorial was originally published as a response to J. Cohen, Science 392:6800, 2026, https://doi.org/10.1126/science.aej0204, republished with permission of AAAS.

## References

[1] Cohen J. 2026. Virologist accused of starting COVID-19 fights funding ban. Science 392:795–796. doi:10.1126/science.aej0204 42166576

